# From perceived typicality of in-vehicle stylistic cues to autonomous vehicle acceptance: the roles of perceived behavioral control and trust

**DOI:** 10.3389/fpsyg.2026.1917454

**Published:** 2026-08-27

**Authors:** Jing Pan, Yang Qin, Yanfei Zhu, Kexin Chen, Mo Chen

**Affiliations:** 1 College of Art and Design, Nanjing Tech University, Nanjing, China; 2 College of Furnishings and Industrial Design, Nanjing Forestry University, Nanjing, China

**Keywords:** autonomous vehicle acceptance, behavioral intention, human–vehicle interaction, in-vehicle experience, perceived behavioral control, perceived typicality of in-vehicle stylistic cues, trust

## Abstract

**Introduction:**

Although autonomous driving technology has advanced, limited public acceptance remains a major barrier. Moving beyond studies focused on isolated design domains, this study examined whether the perceived typicality of stylistic cues across automated driving behavior, vehicle interior design, and human–machine interface (HMI) presentation is associated with autonomous vehicle acceptance.

**Methods:**

The three domain scores were averaged to form the observed In-Vehicle Style Typicality Index (IVSTI). In a stimulus-based online survey, 202 participants evaluated eight stimuli and completed measures of perceived behavioral control (PBC), trust, and behavioral intention. Structural equation modeling was used to examine the proposed associations and indirect effects.

**Results:**

The IVSTI was positively associated with PBC and behavioral intention, but not with trust. The indirect association through PBC was statistically significant, whereas the indirect association through trust alone was not. A significant serial indirect association through PBC and trust was observed in the hypothesized model, although alternative analyses did not establish a unique ordering between PBC and trust. Domain-level analyses showed that all three domains were positively associated with PBC when examined separately, whereas only HMI-style typicality retained a significant unique association when modeled simultaneously.

**Discussion:**

These findings extend autonomous vehicle acceptance research by integrating three conceptually distinct in-vehicle stylistic domains and identifying PBC as an important statistical pathway linking perceived typicality with behavioral intention.

## 1 Introduction

With rapid advances in sensing, artificial intelligence, connectivity, and vehicle automation, autonomous vehicles (AVs) have become an important component of future mobility systems. Their potential to improve road safety, enhance traffic efficiency, and increase travel convenience has attracted substantial attention from both industry and academia (Fagnant and Kockelman, 2015; Huang et al., 2023; Hussain and Zeadally, 2018). However, recent studies indicate that user acceptance of AVs remains limited (Kenesei et al., 2022), suggesting that technological progress alone is insufficient to ensure their successful deployment. Indeed, as AV technologies integrate increasingly diverse functions and interaction modalities, users may face greater uncertainty in understanding and evaluating these systems, which can influence their acceptance (Shojaei and Almansour, 2025). As automation reshapes the relationship between occupants and vehicle control, users must evaluate not only whether an automated system is technically capable but also whether its behavior, in-vehicle environment, and communication are understandable, manageable, and trustworthy.

Research on AV acceptance has drawn extensively on the Theory of Planned Behavior (TPB), the Technology Acceptance Model (TAM), and related extended models. TPB identifies attitudes, subjective norms, and perceived behavioral control (PBC) as determinants of behavioral intention (Ajzen, 1991; Luo et al., 2023), whereas TAM emphasizes perceived usefulness and ease of use (Davis, 1989). Recent AV-adoption studies also highlight trust, perceived risk, perceived enjoyment or hedonic motivation, social influence, and media- and information-related perceptions (Zhang et al., 2024; Kenesei et al., 2025; Vacca and Ko, 2025; Vafaei-Zadeh et al., 2025; Wu and Kim, 2025). Together, these findings indicate that AV acceptance reflects interrelated utilitarian, affective, normative, social, risk-related, and trust-related evaluations rather than a single process.

The present study does not attempt to reproduce the complete structure of TPB, TAM, or an integrated AV-adoption model. Instead, it focuses on PBC and trust as two internal evaluations that are especially relevant to users’ responses to in-vehicle design-related cues. As autonomous driving reduces occupants’ direct involvement in vehicle operation (Lin et al., 2021; Odachowska et al., 2021), users’ perceptions that AV use is understandable, manageable, and within their capabilities become increasingly important. Trust is likewise central because autonomous driving requires users to rely on a technological system under conditions of uncertainty and vulnerability (Kenesei et al., 2022; Kenesei et al., 2025). Although attitudes, subjective norms, perceived usefulness, perceived ease of use, perceived enjoyment, perceived risk, and social influence also contribute to AV adoption, the present study examines a narrower design-related pathway involving perceived capability and willingness to rely on the system. Representative evidence concerning the roles of PBC and trust in AV acceptance is summarized in Supplementary Table 1.

Previous studies have often treated PBC (Cai et al., 2023; Jayaraman et al., 2019; Khastgir et al., 2018; Liu et al., 2019; Naiseh et al., 2025; Xu et al., 2018; Zhang et al., 2021) and trust (Dai et al., 2021; Hamiditehrani et al., 2024; Hollebeek et al., 2023; Jing et al., 2019; Kaye et al., 2020; Pang et al., 2024; Ruan et al., 2024) as parallel predictors or separate mediators of behavioral intention. Comparatively less attention has been paid to whether they may be represented within a theoretically ordered pathway. Users who perceive AV use as understandable and manageable may also report less uncertainty when evaluating whether to rely on the system, providing a theoretical rationale for specifying an association between PBC and trust. However, relatively little is known about whether these internal evaluations are associated with the stylistic information conveyed through the in-vehicle experience.

Users encounter such information through several design domains. Automated driving behavior communicates dynamic cues through speed, acceleration, following distance, lane changing, and responses to surrounding traffic, and has been associated with comfort, trust, intervention behavior, and adaptation to automated systems (Bellem et al., 2018; Lee et al., 2021; Lu et al., 2022; Ma and Zhang, 2021). Vehicle interiors provide relatively stable visual, spatial, and material cues that shape perceptions of the in-vehicle environment. Human–machine interfaces (HMIs), in turn, communicate system status, functions, and intentions through visual and interactional presentation. Research has examined anthropomorphic interfaces (Large et al., 2019), conversational interaction (Ruijten et al., 2018), and HMI design in simulated driving environments (Morra et al., 2019). Collectively, these studies indicate that users’ responses to AVs are associated with multiple forms of dynamic and static design information.

However, automated driving style, interior design style, and HMI style have generally been examined as separate influences. From an occupant-centered perspective, this separation does not fully reflect how an autonomous journey is experienced. While traveling in an autonomous vehicle, occupants encounter the vehicle’s motion and responses, the visual–spatial and material characteristics of the cabin, and the system information presented through the HMI within the same experiential context. These dynamic, environmental, and interactional sources of information collectively contribute to the broader in-vehicle experience. In the present study, therefore, “in-vehicle” refers to the occupant’s experience of being transported by and interacting with the vehicle, rather than only to design elements physically located inside the cabin. Relatively little research has considered stylistic information across these three domains within a common framework.

At the same time, the three domains are qualitatively different: automated driving behavior conveys dynamic behavioral information, interior design conveys visual–spatial and material information, and HMI presentation conveys informational–interactional and visual information. Their inclusion within a common framework does not imply that they are interchangeable manifestations of a single latent attribute or that their coordination has been empirically established. Instead, the present study adopts an occupant-centered in-vehicle experience perspective and focuses specifically on the perceived typicality of stylistic cues within each domain, namely, the extent to which each presented stimulus is judged to exemplify its intended style category. A higher typicality rating indicates stronger perceived correspondence with an intended category rather than stronger preference, greater attractiveness, personal fit, or the superiority of one style over another. To provide an aggregate summary across the three conceptually distinct domains, their domain-level typicality scores are represented by the In-Vehicle Style Typicality Index (IVSTI), an observed composite measure rather than a reflective higher-order latent construct.

The perceived typicality of in-vehicle stylistic cues may be associated with PBC and trust in different ways. Stronger perceived correspondence between the presented cues and their intended style categories may be associated with a stronger perception that the vehicle’s behavior, interior environment, and system information are understandable and manageable. However, perceived typicality does not inherently indicate that a vehicle is safe, dependable, or trustworthy. Aggressive and defensive driving behaviors, for example, may both be perceived as highly typical of their respective categories while conveying different implications for reliance. Trust may therefore depend more strongly on evidence of technical competence, safety performance, operational reliability, and institutional safeguards.

To organize these relationships, the present study draws on the Stimulus–Organism–Response (S–O–R) framework (Mehrabian and Russell, 1974). The perceived typicality of stylistic cues conveyed through automated driving behavior, vehicle interior design, and HMI presentation is positioned as a design-related stimulus perception. PBC and trust represent internal evaluations within the organism component, concerning users’ perceived capability to engage with the technology and their willingness to rely on it, respectively, while behavioral intention represents the response and serves as an indicator of autonomous vehicle acceptance.

Accordingly, this study examines whether the perceived typicality of in-vehicle stylistic cues is associated with PBC, trust, and behavioral intention toward AVs. It further examines the separate indirect associations through PBC and trust and a theoretically ordered serial pathway involving PBC and trust. The study contributes to AV-acceptance research by considering automated driving behavior, vehicle interior design, and HMI presentation within a common framework while preserving their conceptual distinctiveness; by summarizing the domain-level typicality scores through an observed composite index; and by applying the S–O–R framework to examine statistical pathways linking design-related perceptions with autonomous vehicle acceptance.

## 2 Literature review and research hypotheses development

### 2.1 Perceived typicality of in-vehicle stylistic cues

Within the in-vehicle experience from the occupant’s perspective, stylistic information is conveyed through automated driving behavior, vehicle interior design, and human–machine interface (HMI) presentation. Unlike conventional vehicle-styling research, which has primarily focused on exterior appearance or isolated visual attributes, the present study considers both dynamic and static stylistic information encountered by occupants. Such information concerns not only how the in-vehicle environment looks, but also how an autonomous vehicle behaves and communicates with its occupants. This broader perspective has become increasingly relevant as autonomous vehicles evolve into intelligent and interactive environments and as occupants increasingly experience the vehicle as users or passengers rather than as direct operators.

The three domains provide complementary but qualitatively different forms of stylistic information. Automated driving behavior conveys dynamic information about how the vehicle moves and responds to surrounding traffic. Vehicle interior design provides relatively stable visual, spatial, and material cues, whereas the HMI communicates system information and operational status through visual and interactional presentation. Together, they provide coverage of how an autonomous vehicle behaves, how its interior environment is presented, and how the system communicates with its occupants. However, they are not treated as interchangeable manifestations of a single latent construct.

The present study specifically focuses on the perceived typicality of in-vehicle stylistic cues, defined as the extent to which the presented stimuli are perceived as exemplifying their respective intended style categories. Typicality represents a graded judgment of how well an exemplar represents a category rather than a simple decision about category membership (Rosch and Mervis, 1975; Barsalou, 1985; Loken and Ward, 1990). The styles examined within the three domains therefore represent distinct categories rather than higher and lower levels of a common stylistic continuum. A higher typicality rating indicates stronger perceived correspondence with an intended category rather than stronger preference, greater attractiveness, or the superiority of one style over another. The following subsections review the three stylistic-cue domains and their relevance to users’ evaluations of autonomous vehicles.

#### 2.1.1 Automated driving style

Automated driving style represents the dynamic behavioral domain examined in the present study. In conventional driving research, driving style generally refers to relatively stable patterns of driving behavior shaped by individual characteristics, social context, and situational conditions (Elander et al., 1993). Sagberg et al. (2015) distinguished between global driving styles, which reflect broader behavioral tendencies and motivations, and specific driving styles, which are expressed through observable behaviors such as speeding, hard braking, or tailgating. Although terminology and classification criteria vary across studies, aggressive and defensive driving (the latter sometimes termed conservative driving) are frequently examined as contrasting behavioral categories (Ma and Zhang, 2021).

In autonomous driving contexts, driving style no longer refers only to the habitual behavior of a human driver. It also describes the system-generated patterns of vehicle control perceived by occupants. These patterns are conveyed through vehicle speed, acceleration and deceleration, following distance, lane-changing and overtaking behavior, responses to hazards, and the smoothness or assertiveness of longitudinal and lateral control. Automated driving style therefore provides perceptible behavioral cues from which occupants may form expectations about how the vehicle is likely to respond in different traffic situations.

Aggressive automated driving is generally characterized by higher speeds, more rapid acceleration and deceleration, shorter following distances, and more assertive responses to surrounding traffic. Defensive automated driving, in contrast, emphasizes risk avoidance, smoother control, larger safety margins, and more cautious responses. These characteristics distinguish contrasting behavioral categories rather than favorable and unfavorable levels of a common attribute. They may also provide occupants with information concerning the apparent caution, responsiveness, and predictability of the automated vehicle.

Previous studies have demonstrated that automated driving style is associated with users’ comfort, trust, anxiety, and adaptation to automated systems. Ma and Zhang (2021) found that greater correspondence between users’ preferred driving characteristics and the behavior of an automated vehicle was associated with stronger trust and fewer takeover behaviors. Lee et al. (2021) compared defensive, normal, and aggressive automated driving styles in a simulated environment and found that defensive driving was more likely to elicit accelerator input, whereas aggressive driving was more likely to induce braking responses. These behavioral responses suggest that users may interpret defensive driving style as overly cautious and an aggressive driving style as requiring greater intervention, thereby illustrating how system behavior influences users’ evaluations. Lu et al. (2022) further showed that automated driving style affected users’ adaptation from active drivers to passive passengers by moderating the relationship between state anxiety and role adaptation.

Collectively, these findings indicate that automated driving style is not merely a technical configuration of vehicle movement but a distinct source of dynamic behavioral information through which occupants may form expectations about an autonomous vehicle’s caution, responsiveness, and predictability.

#### 2.1.2 Interior design style

Interior design style represents the spatial and environmental domain examined in the present study. As automotive functions have become increasingly sophisticated, users’ attention to vehicle interiors has expanded beyond basic functionality and ergonomics to include atmosphere, visual character, spatial quality, and material expression (Jindo and Hirasago, 1997). With increasing vehicle automation and reduced occupant involvement in the driving task, the interior is expected to support a broader range of passenger activities and experiences, including entertainment, communication, work, and relaxation (Riegler et al., 2021).

This transformation is particularly relevant to autonomous vehicles, as reduced involvement in direct vehicle operation creates new possibilities for seating configurations, spatial arrangements, information displays, lighting systems, and material applications (Kim et al., 2023). The vehicle interior consequently serves not only as the physical environment in which automated mobility occurs but also as an important source of perceptual information concerning the intended character of the in-vehicle experience.

Interior design style is conveyed through elements including color schemes, materials, lighting, spatial layout, surface treatment, and seating configuration. These elements contribute to users’ perceptions of openness, orderliness, comfort, refinement, sportiness, and technological sophistication. For example, modern minimalist interiors commonly employ restrained forms, clean lines, uncluttered surfaces, and relatively neutral color schemes. Futuristic technology-oriented interiors may feature digital surfaces, cool-toned lighting, streamlined forms, and visually prominent technological elements. Natural and inviting interiors may use warmer materials, softer forms, and colors associated with natural environments, whereas dynamic sporty interiors may employ stronger contrasts, angular forms, and performance-oriented visual features.

Previous research has shown that users can distinguish vehicle interior styles through perceptual attributes such as luxury, refinement, orderliness, and sportiness (Jindo and Hirasago, 1997). Spatial organization, including seating arrangement and orientation, may also influence how occupants perceive the character, comfort, and suitability of the in-vehicle environment. Studies of shared autonomous vehicles have further suggested that seating configurations can affect passengers’ expected comfort, trust, and sense of control, indicating that interior layout is an important component of the anticipated vehicle experience (Aasvik et al., 2025).

Interior design style therefore constitutes a distinct source of visual, spatial, and material information through which occupants may form impressions of the character, organization, and atmosphere of the in-vehicle environment.

#### 2.1.3 HMI style

HMI style represents the informational and interactional domain examined in the present study. As automotive HMIs continue to evolve, users’ evaluations of interfaces extend beyond the efficiency of information transmission to include the stylistic, affective, and personalized qualities of the interaction experience (Hartwich et al., 2021; El Jouhri et al., 2023). In the present study, automotive HMI style concerns how system information, interactive functions, and interface characteristics are visually and structurally presented within an autonomous vehicle.

Automotive HMIs communicate system status, navigation information, warnings, automated driving functions, and available user controls. As autonomous driving reduces occupants’ direct involvement in vehicle operation, the HMI becomes an increasingly important channel through which users understand the system, maintain awareness of the driving situation, and interact with automated functions. The cognitive effectiveness of in-vehicle information presentation may also depend on the interaction between driving-task demands and interface information load, indicating that HMI design should balance information richness with users’ cognitive-processing capacity (Li et al., 2026).

Existing research suggests that HMI characteristics can influence users’ trust, situation awareness, perceived usability, and broader evaluations of automated systems (El Jouhri et al., 2023; Ma et al., 2021; Yekta and Schöning, 2024). Interface features that communicate system status and intentions clearly may help users interpret automated functions and form more informed judgments about the system. In a broader human–computer interaction context, Hasan and Ahmed (2007) found that interface style affected users’ perceptions and behavioral intentions, indicating that the organization and presentation of interactive functions can shape technology acceptance.

Visual and affective characteristics also contribute to users’ perceptions of HMI style. Ren et al. (2020) showed that different combinations of color and visual expression in automotive interfaces produced different affective evaluations. These findings indicate that HMI design communicates more than functional information; it also provides stylistic cues that may contribute to users’ broader impressions of the vehicle and its interaction system.

Flat and skeuomorphic interfaces represent two contrasting approaches to visual and interactional presentation. Flat design typically emphasizes simplicity, two-dimensional forms, limited ornamentation, and visual clarity, whereas skeuomorphic design incorporates realistic textures, shadows, depth, and references to familiar physical objects. These stylistic differences may influence how users interpret interface elements and perceive the visual and interactional qualities of the system.

HMI style therefore constitutes a distinct source of informational, visual, and interactional cues through which users may interpret the automated system and the way it communicates with them. Together with automated driving style and interior design style, it forms one of the three stylistic domains considered in the present study.

### 2.2 Perceived behavioral control

Perceived behavioral control (PBC) originates from the Theory of Planned Behavior (TPB), which provides a framework for understanding the cognitive determinants of behavioral intention (Ajzen, 1991). TPB proposes that behavioral intention is shaped by three principal constructs: attitudes, subjective norms, and PBC (Castanier et al., 2013). PBC refers to an individual’s perception of the ease or difficulty of performing a given behavior. In the context of autonomous vehicles (AVs), it reflects users’ confidence in their ability to understand and use automated functions and their perception that using an AV is within their capabilities or control (Buckley et al., 2018). In the present study, PBC primarily concerns users’ perceived capability to use AVs rather than their retention of direct physical control over vehicle operation.

PBC has consistently been identified as an important factor in AV acceptance. When individuals perceive AV use as manageable and within their capabilities, they are generally more likely to report stronger intentions to use or adopt the technology. For example, Molinillo et al. (2024) found that PBC was positively associated with users’ behavioral intention toward autonomous busses. Liu et al. (2022) similarly reported that greater perceived control over Level 3 automated vehicles was associated with stronger intentions to use them. Luo et al. (2023) further showed that PBC was directly associated with willingness to adopt shared autonomous vehicles and mediated the associations of government policies, technological interest, and subjective norms with behavioral intention. These findings suggest that PBC may function as a relatively proximal psychological evaluation through which broader perceptions of AV technology are associated with acceptance intentions.

Perceived control may also be statistically associated with trust. Evidence from other technology-mediated contexts suggests that individuals who perceive greater ability to understand and manage their interaction with a system or information source may also report greater trust. For instance, Sembada and Koay (2021) found that perceived behavioral control was related to trust in social-media purchasing contexts, while Deng et al. (2021) reported a positive association between PBC and trust in online health information. Although these findings do not establish the same relationship in autonomous driving, they provide a theoretical basis for examining an association between PBC and trust under conditions of technological uncertainty. When interaction with an automated system appears more comprehensible and manageable, users may also report lower uncertainty when evaluating whether to rely on it.

Relatively little research has examined whether the perceived typicality of stylistic information conveyed through the in-vehicle experience is associated with users’ PBC. When the stylistic cues conveyed through automated driving behavior, vehicle interior design, and HMI presentation are perceived as closely corresponding to their intended categories, the vehicle’s behavior, in-vehicle environment, and system information may appear more readily interpretable to users. Although such perceptions do not necessarily alter users’ objective ability to use an AV, they may be associated with a stronger subjective sense that AV use is understandable, manageable, and within their capabilities. PBC may therefore help account for the association between the perceived typicality of in-vehicle stylistic cues and behavioral intention, while the possible association between PBC and trust remains to be examined empirically.

### 2.3 Trust

Trust has emerged as a central psychological factor in research on the acceptance of automated systems and artificial intelligence technologies. It is commonly defined as an attitude reflecting an individual’s willingness to rely on an agent under conditions of uncertainty and vulnerability, based on positive expectations that the agent will support the individual’s goals (Lee and See, 2004). In the context of autonomous driving, trust reflects users’ willingness to rely on an automated vehicle despite their limited ability to directly verify or control all aspects of its operation.

Trust is particularly important because the transfer of driving functions from human drivers to automated systems requires users to evaluate whether the vehicle is sufficiently capable, predictable, safe, and dependable to justify reliance. Choi and Ji (2015) identified system transparency, technical competence, and situation management as important foundations of trust and demonstrated that trust plays a substantial role in explaining users’ intentions to adopt autonomous vehicles. Appropriate trust is also important for safe human–automation interaction: insufficient trust may result in rejection or underuse, whereas excessive trust may lead to overreliance or misuse of the system (Nordhoff et al., 2023).

Trust in automation is neither uniform nor static. Hoff and Bashir (2015) distinguished among dispositional trust, situational trust, and learned trust. Dispositional trust reflects an individual’s general tendency to trust automation, situational trust is shaped by contextual conditions, and learned trust develops through information and prior interaction with a system. The present study is primarily concerned with users’ evaluative trust in autonomous vehicles, as reflected in their judgments of system safety, safeguards, and overall trustworthiness, rather than with a stable tendency to trust technology.

A substantial body of research has identified trust as an important predictor of autonomous vehicle acceptance and behavioral intention. Kenesei et al. (2022) found that performance-based trust significantly influenced users’ intentions to adopt autonomous driving. Kaur and Rampersad (2018) similarly identified trust, reliability, safety, privacy, and performance expectancy as important factors in autonomous vehicle adoption. Benleulmi and Ramdani (2022) further demonstrated that trust operates alongside instrumental motivations, such as efficiency and cost savings, and symbolic motivations, such as personalization and social status, in shaping behavioral intention. Collectively, these findings indicate that users are more likely to consider adopting autonomous vehicles when they perceive the technology as safe, dependable, and capable of acting in their interests.

Trust has also been examined as a psychological mechanism through which users’ evaluations of autonomous vehicles are associated with acceptance. Man et al. (2020) found that trust was directly associated with intention to use autonomous vehicles and was also related to users’ attitudes through perceived usefulness. Lu et al. (2022) further indicated that trust was involved in users’ adaptation to autonomous driving through its relationships with situational awareness and state anxiety. These findings suggest that trust may help link users’ evaluations of an automated vehicle with their willingness to rely on and use the technology.

Trust may be associated partly with perceptual cues provided by the in-vehicle experience. Automated driving behavior may communicate whether the system appears predictable and capable of responding appropriately to traffic conditions. Interior design may provide contextual cues related to orderliness and technological quality, whereas HMI design may communicate system status, functionality, and operating logic. By drawing on these distinct dynamic and static cues, users may develop broader judgments regarding the dependability and trustworthiness of an autonomous vehicle. However, the perceived typicality of stylistic cues does not itself demonstrate that the vehicle is technically competent, operationally reliable, or safe. Trust may therefore also depend on evaluations of technical competence, safety assurance, and institutional safeguards that are not fully conveyed through stylistic cues alone.

Trust may also be related to perceived behavioral control. When users believe that they are capable of understanding and using an autonomous vehicle, uncertainty associated with the technology may be reduced, enabling them to evaluate and rely on the system with greater confidence. PBC may therefore provide a psychological basis for trust by making interaction with the autonomous vehicle appear more comprehensible and manageable. Evidence from other technology-mediated contexts has similarly suggested that perceived control can support trust formation (Deng et al., 2021; Sembada and Koay, 2021).

Overall, trust represents users’ willingness to rely on autonomous vehicles on the basis of perceived safety, dependability, and overall trustworthiness. It may constitute one possible statistical pathway between the perceived typicality of in-vehicle stylistic cues and behavioral intention, while the possible association between PBC and trust provides a theoretical basis for the serial statistical pathway proposed in the following section.

### 2.4 Research model and hypotheses

#### 2.4.1 Integrating the focal constructs through the S–O–R framework

The Stimulus–Organism–Response (S–O–R) framework has recently been applied in technology-mediated consumer research (Shojaei et al., 2026a) and provides the overarching logic for integrating the constructs reviewed in the preceding sections. It proposes that environmental stimuli are associated with individuals’ internal cognitive and affective evaluations, which are, in turn, related to behavioral responses (Mehrabian and Russell, 1974). In the present study, the automated driving videos, vehicle interior renderings, and HMI images constitute the stimulus materials, while the perceived typicality of the stylistic cues conveyed through these materials is treated as the stimulus-related variable in the empirical model. Although perceived typicality is a subjective judgment rather than an objective property of the materials, it directly reflects participants’ perception of external stylistic attributes and functions as a proximal input to subsequent internal evaluations. Perceived behavioral control (PBC) and trust represent internal evaluations, whereas behavioral intention represents the response.

The three stylistic domains convey different forms of design-related information. Automated driving style provides dynamic behavioral cues, interior design style provides visual–spatial and material cues, and HMI style provides informational–interactional and visual cues. As discussed in Section 2.1, these domains are conceptually distinct and are not treated as reflective manifestations of a common higher-order latent construct. Instead, the present study evaluates the extent to which the stimuli within each domain are perceived as exemplifying their respective intended style categories.

PBC and trust are selected as the focal organism variables because they capture two complementary evaluations that are especially relevant to autonomous driving. PBC concerns whether users perceive AV use as understandable, manageable, and within their capabilities, whereas trust concerns whether users are willing to rely on an automated system under conditions of uncertainty. PBC therefore reflects perceived capability to engage with the technology, while trust reflects willingness to depend on it.

The proposed model does not attempt to reproduce the complete structure of the Theory of Planned Behavior, the Technology Acceptance Model, or a comprehensive model of AV adoption. Previous research has shown that attitudes, subjective norms, social influence, perceived usefulness, perceived ease of use, perceived enjoyment, and perceived risk also contribute to AV acceptance. These variables capture broader utilitarian, affective, normative, social, and risk-related evaluations (Almansour et al., 2025). The present study instead focuses on a narrower design-related pathway linking the perceived typicality of in-vehicle stylistic cues with PBC, trust, and behavioral intention.

#### 2.4.2 Research hypotheses and proposed model

Building on the S–O–R framework, the present study examines whether the perceived typicality of stylistic cues conveyed through automated driving behavior, vehicle interior design, and HMI presentation is associated with PBC, trust, and behavioral intention toward AVs. Greater perceived typicality indicates that the presented stimuli are judged to correspond more strongly to their respective intended style categories, and their behavioral, visual–spatial, and informational–interactional characteristics may therefore be more discernible to users.

However, the perceived typicality of in-vehicle stylistic cues does not inherently indicate that a style is favorable, desirable, safe, or trustworthy. The styles examined in this study represent distinct categories rather than higher and lower levels of a common positive attribute. For example, aggressive and defensive driving behaviors may both be perceived as highly typical of their respective categories while conveying different implications for users’ evaluations. The relationships between perceived typicality and the focal variables are therefore specified as non-directional hypotheses:

*H1a*: The perceived typicality of in-vehicle stylistic cues is associated with perceived behavioral control.

*H1b*: The perceived typicality of in-vehicle stylistic cues is associated with trust in autonomous vehicles.

*H1c*: The perceived typicality of in-vehicle stylistic cues is associated with behavioral intention toward autonomous vehicles.

As discussed in section 2.2, PBC may constitute a statistical pathway through which users’ evaluations of in-vehicle stylistic information are associated with behavioral intention. When the stylistic cues conveyed through automated driving behavior, vehicle interior design, and HMI presentation are perceived as strongly corresponding to their intended categories, these aspects of the in-vehicle experience may appear easier to interpret. This, in turn, may be associated with a stronger perception that AV use is manageable and within users’ capabilities, which may relate to stronger behavioral intention. Accordingly:

*H2*: The perceived typicality of in-vehicle stylistic cues is indirectly associated with behavioral intention through perceived behavioral control.

Trust may constitute a second indirect pathway. Stylistic information may contribute to users’ impressions of the vehicle’s behavior, quality, and system character, which may be relevant to their willingness to rely on the automated system. Nevertheless, perceived typicality does not itself provide direct evidence of technical competence, safety performance, or operational reliability. The direction and strength of the indirect association through trust are therefore treated as empirical questions rather than predetermined outcomes. Accordingly:

*H3*: The perceived typicality of in-vehicle stylistic cues is indirectly associated with behavioral intention through trust.

The preceding discussion further suggests that PBC and trust may be represented within a theoretically ordered serial pathway. Users who perceive AV use as understandable and manageable may also report more favorable judgments regarding whether to rely on the system. In the specified model, trust is, in turn, associated with behavioral intention. Accordingly:

*H4*: There is a serial indirect association between the perceived typicality of in-vehicle stylistic cues and behavioral intention through perceived behavioral control and trust, in that order.

The proposed model includes the direct associations specified in H1a–H1c and the indirect associations specified in H2–H4. In the empirical analysis, the aggregate level of perceived typicality across the three stylistic domains is represented by the In-Vehicle Style Typicality Index (IVSTI), an observed composite measure rather than a reflective higher-order latent construct.

**FIGURE 1:**
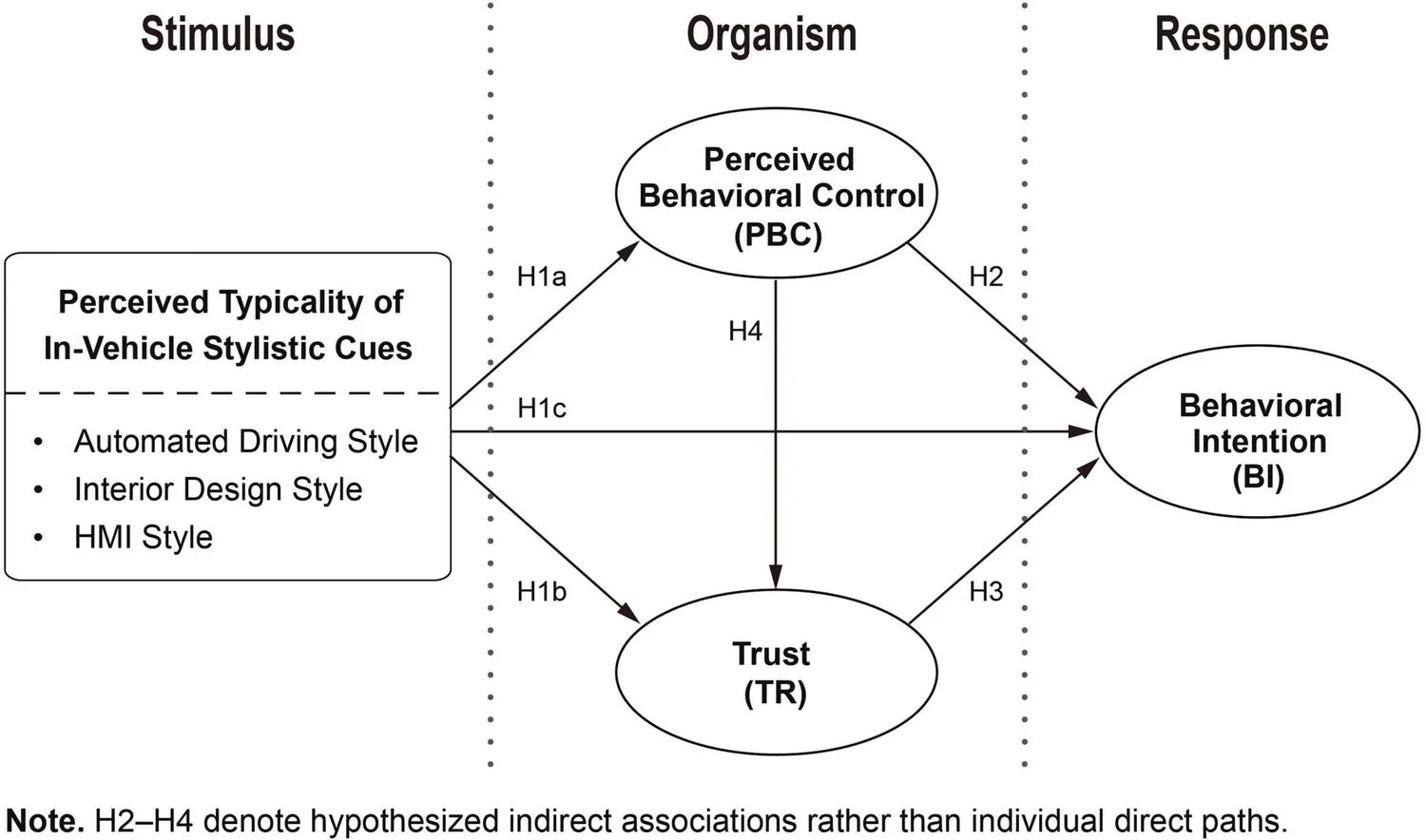
Proposed research model.

Because the study uses cross-sectional self-report data, the proposed ordering represents a theoretically specified statistical structure rather than evidence of temporal precedence or causality. The proposed research model is presented in Figure 1.

## 3 Methodology

### 3.1 Pilot study: stimulus screening

Before the formal survey, a pilot study was conducted to screen candidate vehicle interior and HMI images and identify recurrent and interpretable patterns of perceived stylistic similarity. Consistent with multiple-sorting and picture-sorting approaches, the pilot study was designed as an exploratory procedure for examining how participants organized the images according to perceived similarity and for identifying groupings that could inform subsequent stimulus development. A multiple sorting procedure was implemented as an unlabeled repeated picture-sorting task, in which participants sorted the same set of stimuli across several rounds with different prescribed numbers of groups (Canter et al., 1985; Rugg and McGeorge, 1997; Lobinger and Brantner, 2020).

Automated driving-style stimuli were not included in the sorting task because the aggressive and defensive automated driving categories were operationalized on the basis of behavioral characteristics and parameter ranges established in prior research. The development and presentation of these automated driving-style stimuli are described in section 3.2.3.

First, automotive interior and HMI images representing different stylistic characteristics were collected from online sources. The candidate interior design styles included luxurious classic, modern minimalist, futuristic technology-oriented, natural and inviting, dynamic sporty, and retro styles. The candidate HMI styles included flat design, skeuomorphism, light skeuomorphism, three-dimensional design, and gamification. These preliminary labels were used only to guide the construction of the candidate image pool and were not shown to participants during the sorting task. Ten candidate images were selected for each of the two image sets to provide sufficient coverage of the candidate stylistic characteristics.

Twenty participants completed separate sorting tasks for the vehicle interior and HMI image sets. For each image set, participants completed three sorting rounds according to perceived stylistic similarity. In the first round, the images were sorted into two groups; in the second round, they were sorted into three groups; and in the third round, they were sorted into four groups. Although the number of groups was specified in each round, group composition was determined by the participants. The group membership of each image was recorded after each round.

Following the exploratory logic of the sorting procedure, the resulting patterns were reviewed descriptively across participants and rounds. Attention was directed to three related aspects:

1. Co-classification recurrence: whether particular images were repeatedly placed together across the sorting tasks;
2. Pattern stability: whether similar image groupings reappeared across participants and sorting rounds;
3. Interpretive coherence: whether the images within recurrent groupings shared recognizable visual, spatial, or interactional characteristics.

These aspects were used to identify recurrent and interpretable patterns of image organization that could provide a basis for subsequent stimulus development. After recurrent sorting patterns had been identified, the researchers reviewed the shared visual, spatial, or interactional characteristics of each cluster and assigned descriptive labels retrospectively using terminology commonly employed in automotive and interface design. The labels were therefore used to describe the groupings emerging from participants’ sorting patterns and were not supplied to participants as predetermined response categories.

Based on the recurrence, stability, and interpretive coherence of the observed sorting patterns, four interior design styles—modern minimalist, futuristic technology-oriented, natural and inviting, and dynamic sporty—and two HMI styles—flat and skeuomorphic—were retained as the target style categories for subsequent stimulus development. These categories were retained because their corresponding image groupings were recurrent, comparatively consistent, and interpretable within the pilot sample. They were treated as target categories for stimulus development within the candidate image pool rather than as an exhaustive or universally applicable taxonomy of vehicle interior and HMI styles. The final interior and HMI stimuli were subsequently created by the researchers on the basis of these retained category definitions and were not independently pretested with a separate sample before the main study.

### 3.2 Formal study

#### 3.2.1 Participants

Prior to questionnaire distribution, G*Power software was used to estimate the required sample size. With an effect size of 0.15, a significance level of 0.05, and a statistical power of 0.95, the analysis indicated that approximately 119 participants were required.

Participants were recruited through social media advertisements that specified the purpose of the study, the estimated completion time, compensation, and eligibility criteria. The eligibility criteria included no history of neurological or psychiatric disorders, abstinence from alcohol consumption before participation, and normal or corrected-to-normal vision.

Data were collected through the online survey platform SurveyPlus from October 12 to October 15, 2024. A total of 204 participants responded to the survey. During data-quality screening, two responses were excluded because they selected the same response option across nearly all questionnaire items, indicating mechanically repetitive rather than attentive responding. The final analytical sample therefore comprised 202 participants. The participants ranged in age from 18 to 60 years and were predominantly young adults.

All participants provided informed consent before beginning the study and received monetary compensation for their participation. The study protocol was reviewed and approved by the Ethics Review Committee of Nanjing Tech University (Approval No. NJTECH-1-12). The demographic characteristics of the participants are presented in Table 1.

**TABLE 1:** Demographic characteristics of the participants.

Characteristics		N	Percentage
Gender	Male	95	47.03%
	Female	107	52.97%
Age	18–24 years	141	69.80%
	25–30 years	47	23.26%
	31–40 years	6	2.97%
	41–50 years	7	3.47%
	51–60 years	1	0.50%
	More than 61 years	0	0.00%
Highest education level	High school or below	4	1.98%
	Associate degree	6	2.97%
	Bachelor’s degree	121	59.90%
	Master’s degree or above	71	35.15%
Years of driving experience	Under 1 year	118	58.42%
	1–3 years	61	30.20%
	3–5 years	12	5.94%
	5–10 years	7	3.47%
	Over 10 years	4	1.98%

#### 3.2.2 Measurement items and variable construction

The questionnaire consisted of three sections. The first section collected participants’ demographic information, including gender, age, occupation, highest educational level, and years of driving experience. The second section assessed the perceived typicality of stylistic cues conveyed through automated driving behavior, vehicle interior design, and human–machine interface (HMI) presentation. The third section measured trust, perceived behavioral control (PBC), and behavioral intention toward autonomous vehicles.

The perceived typicality of in-vehicle stylistic cues was assessed using eight category-specific items corresponding to the eight presented stimuli. Each item measured the extent to which a stimulus was perceived as exemplifying its respective intended style category. The two automated driving-style items corresponded to the aggressive and defensive driving stimuli and were developed with reference to Ma and Zhang (2021). The four interior design-style items corresponded to the modern minimalist, futuristic technology-oriented, natural and inviting, and dynamic sporty interior stimuli and were developed with reference to Jindo and Hirasago (1997). The two HMI-style items corresponded to the flat and skeuomorphic interface stimuli and were developed with reference to prior work on interface representation styles, including Lee et al. (2024). A higher item score indicated stronger perceived correspondence between a presented stimulus and its intended style category. These ratings assessed category typicality rather than participants’ preference for a particular style.

The perceived typicality of in-vehicle stylistic cues was operationalized at both the domain and overall levels. Automated driving-style typicality was calculated as the mean of the aggressive and defensive driving-style ratings. Interior design-style typicality was calculated as the mean of the four interior-style ratings, and HMI-style typicality was calculated as the mean of the flat and skeuomorphic interface ratings. The three domain-level scores were subsequently averaged, with equal weight assigned to each domain, to derive an observed composite measure termed the In-Vehicle Style Typicality Index (IVSTI). This two-stage calculation ensured that each stylistic domain received equal representation in the overall index despite the different numbers of stimuli used within the three domains. Equal weighting was used as a design-based aggregation rule to give each domain equal representation in the index; it was not intended to imply that the three domains have identical domain-specific associations with the outcome variables. The IVSTI therefore summarizes each participant’s typicality judgments across the complete set of stylistic stimuli; it does not represent the stylistic coherence of a single integrated vehicle configuration.

Accordingly, higher IVSTI scores indicated stronger average correspondence between the presented stimuli and their respective intended style categories. The IVSTI was treated as an observed composite index rather than a reflective higher-order latent construct and did not represent participants’ style preferences or perceived design quality.

Trust, PBC, and behavioral intention were each assessed using three-item reflective scales. The trust and PBC items were adapted from Molinillo et al. (2024), whereas the behavioral-intention items were adapted from Luo et al. (2023). The wording of the items was contextualized for the AV setting while retaining the conceptual content of the original measures. The complete item wording and sources are presented in Table 2.

**TABLE 2:** Measurement items and sources.

Variable		Question	Description of measurement items	Source
Perceived typicality (IVSTI components)	Driving style	DS1	I consider the driving style of the automated vehicle shown above to be aggressive, as reflected in its higher speed, tendency to speed, and shorter distances from surrounding road users or objects.	Ma and Zhang, 2021
		DS2	I consider the driving style of the automated vehicle shown above to be defensive, as reflected in its lower speed, smoother acceleration and deceleration, and greater distances from surrounding road users or objects.	
	Interior design style	IS1	I think the interior design style of the automated vehicle shown in the image is modern minimalist.	Jindo and Hirasago, 1997
		IS2	I think the interior design style of the automated vehicle shown in the image is futuristic technology-oriented.	
		IS3	I think the interior design style of the automated vehicle shown in the image is natural and inviting.	
		IS4	I think the interior design style of the automated vehicle shown in the image is dynamic sporty.	
	HMI style	HMI1	I think the HMI style shown in the image is flat.	Lee et al., 2024
		HMI2	I think the HMI style shown in the image is skeuomorphic.	
Trust	–	TR1	I believe that AVs provide a robust and safe environment for travel.	Molinillo et al., 2024
	–	TR2	I believe that AV providers have adequate safeguards to protect me from liability for damages for which I am not responsible.	
	–	TR3	Overall, AVs are trustworthy.	
PBC	–	PBC1	I believe I am capable of using AVs.	Molinillo et al., 2024
	–	PBC2	I do not think I would have difficulty using AVs.	
	–	PBC3	It would be easy for me to use AVs.	
BI	–	BI1	I will try to use AVs.	Luo et al., 2023
	–	BI2	I would consider AVs a viable mode of travel in the future.	
	–	BI3	I would encourage people around me to use AVs.	

The questionnaire was administered in Chinese. The English item wording presented here is a translation of the administered items.

All measures used five-point response formats. The style-typicality items were administered using category-specific instructions and response anchors appropriate to stimulus evaluation, whereas the trust, PBC, and behavioral-intention items were rated using agreement-based Likert scales ranging from 1 (“strongly disagree”) to 5 (“strongly agree”). Higher scores indicated greater perceived typicality or stronger agreement with the corresponding statement, as applicable.

#### 3.2.3 Study procedure

**FIGURE 2:**
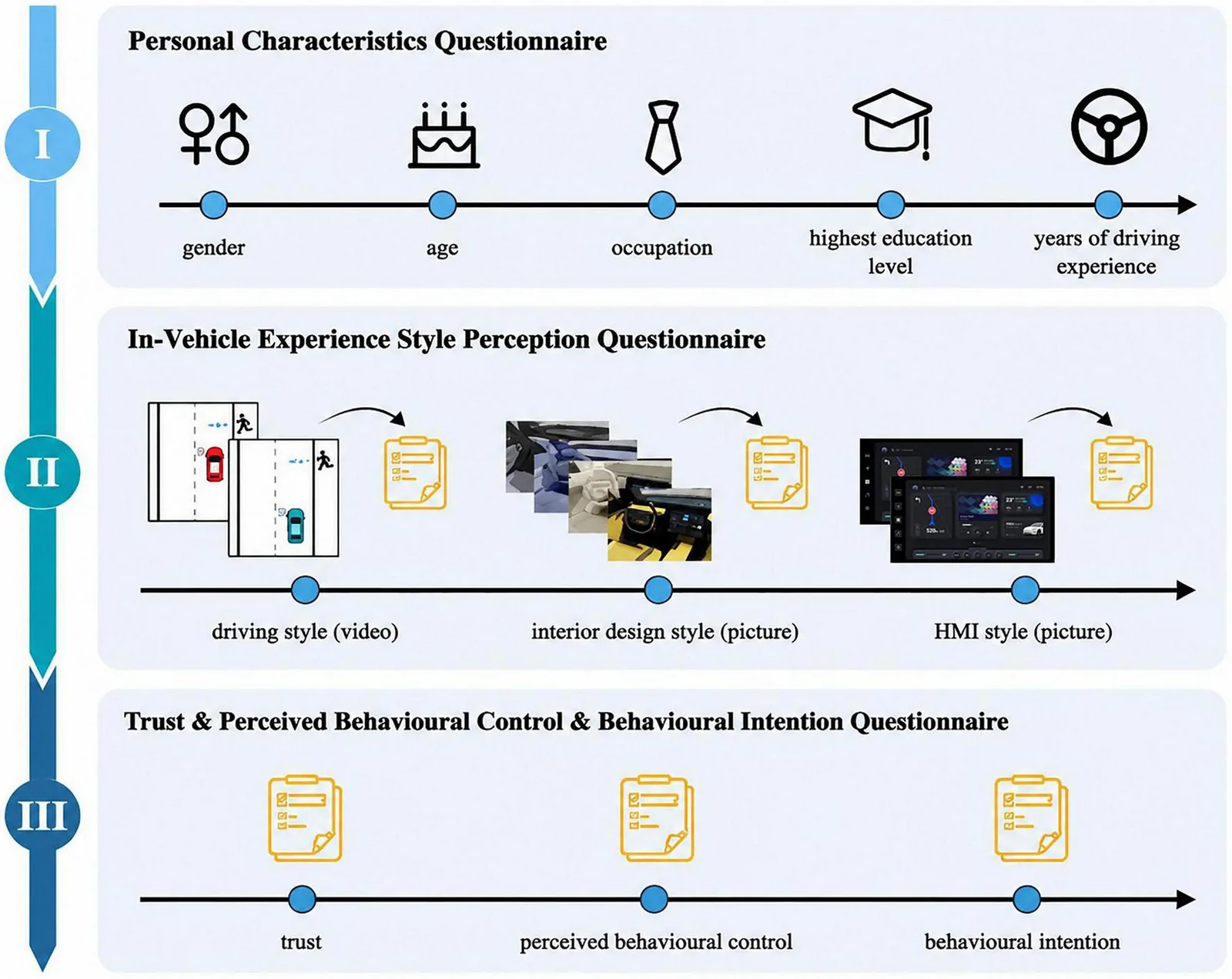
Study procedure.

The study procedure is illustrated in Figure 2 and comprised three stages.

In the first stage, participants received a brief introduction to the purpose and procedure of the study. They were informed that participation was anonymous, that their responses would be used only for research purposes, and that there were no correct or incorrect answers. After providing informed consent, participants completed the first questionnaire section, which collected demographic and driving-related information, including gender, age, occupation, highest educational attainment, and years of driving experience.

In the second stage, participants viewed and evaluated eight stimuli covering the three in-vehicle stylistic domains: two automated driving videos, four vehicle interior renderings, and two HMI images. All participants were exposed to all eight stimuli. The presentation order of the stimuli was randomized for each participant to reduce potential order and carryover effects. Each stimulus was presented on a separate page and was followed by a category-specific item assessing the extent to which it exemplified its intended style category.

For the automated driving stimuli, participants were informed that the depicted vehicle represented a fully automated vehicle corresponding to SAE Level 5 and was assumed to perform the entire driving task in the presented scenarios, including responses to hazardous situations. The videos were created using SCANeR Studio and depicted a typical urban road environment comprising a four-lane, two-way road, moderate traffic density, roadside buildings, and pedestrians. Vehicle-dynamic parameters, including average speed, acceleration, deceleration, and safe following time, were specified with reference to previous studies of aggressive and defensive driving styles (Deffenbacher et al., 2003; Hill et al., 2015; Ma and Zhang, 2021). The specific parameter settings are presented in Table 3.

**TABLE 3:** Driving parameters of the aggressive and defensive autonomous vehicles.

Parameters of AV	Aggressive AVs	Defensive AVs
Average speed (km/h)	85.00	72.00
Acceleration (m/s^2^)	1.84	0.50
Deceleration (m/s^2^)	−3.40	−1.45
Safe following time (s)	0.90	3.50

**FIGURE 3:**
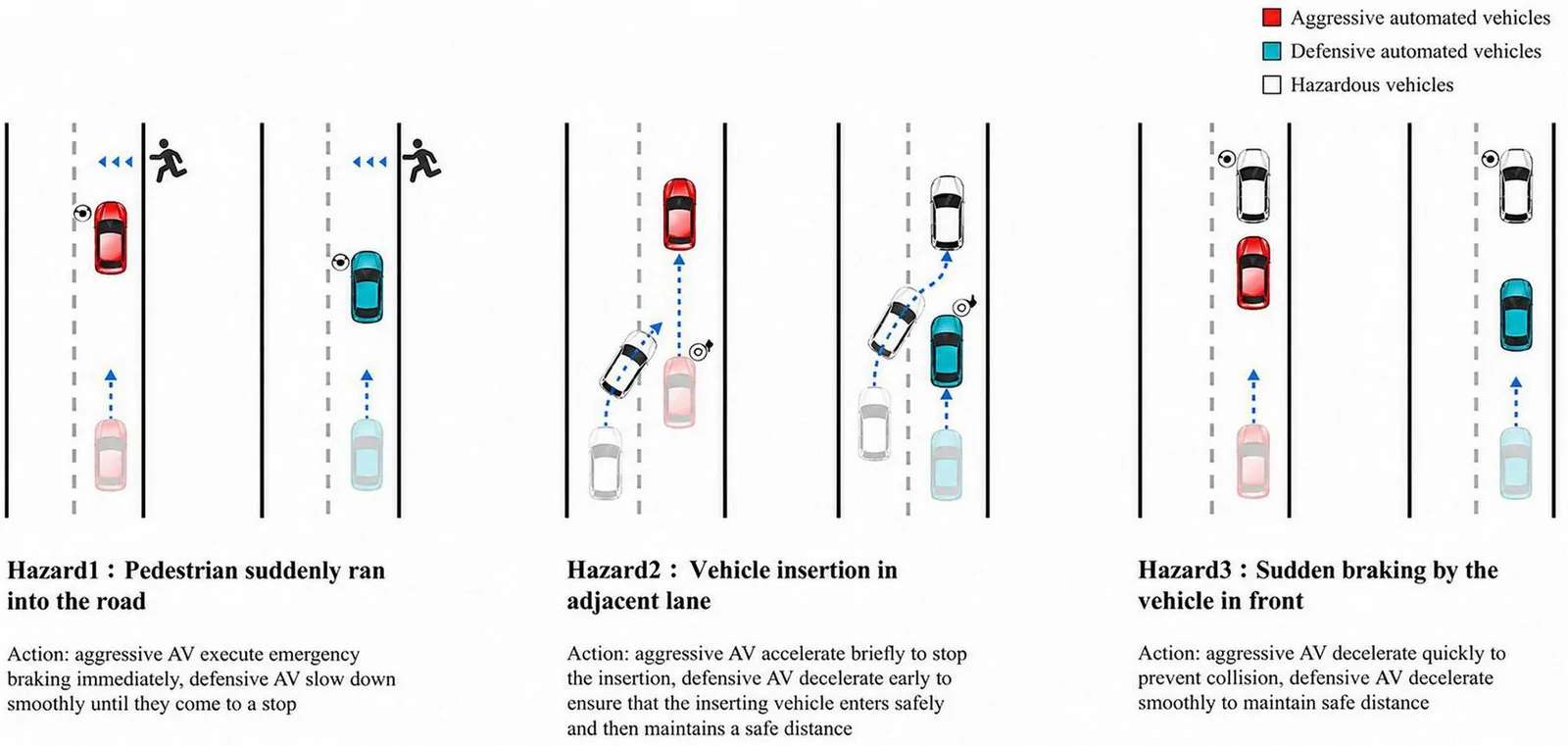
Schematic representation of the hazardous driving scenarios.

The simulated driving task included both normal and hazardous scenarios, as summarized in Table 4. The hazardous scenarios were developed with reference to reported AV accidents and adapted to the context of the present study (Favarò et al., 2018; Ma and Zhang, 2021). Figure 3 illustrates the hazardous scenarios. Based on the specified vehicle-dynamic parameters and scenario configurations, two videos representing aggressive and defensive automated driving styles were produced. Participants were instructed to imagine themselves as occupants of the automated vehicle while viewing each video. After each video, they rated the extent to which the presented driving behavior exemplified its intended style category.

**TABLE 4:** Driving scenarios used in the autonomous driving task.

Scenarios		Aggressive automated vehicle	Defensive automated vehicle
Normal 1	Straight-road cruising	Maintain a high traveling speed and keep a small safe distance from the vehicle in front of you.	Maintain a steady driving speed and keep a large safe distance from the vehicle in front of you.
Hazard 1	A pedestrian suddenly enters the road	When the system detects a pedestrian, it immediately executes emergency braking and accelerates to resume traveling as soon as the pedestrian passes by.	The system detects a pedestrian and decelerates smoothly until it comes to a stop, then slowly accelerates and resumes traveling after the pedestrian passes.
Normal 2	Crossing an intersection after the traffic signal turns green	Accelerate through the intersection as soon as the light turns green.	Accelerate smoothly through the intersection when the light turns green.
Normal 3	Straight-road cruising	Maintain a high traveling speed and keep a small safe distance from the vehicle in front of you.	Maintain a steady driving speed and keep a large safe distance from the vehicle in front of you.
Hazard 2	A vehicle cuts in from an adjacent lane	The vehicle prioritizes maintaining its speed and lane position, provides limited space for the merging vehicle, and may briefly accelerate to discourage the cut-in maneuver.	The vehicle slows down in advance, yields sufficient space to the merging vehicle, and restores a safe following distance after the vehicle enters the lane.
Normal 4	Straight-road cruising	Maintain a high traveling speed and keep a small safe distance from the vehicle in front of you.	Maintain a steady driving speed and keep a large safe distance from the vehicle in front of you.
Normal 5	Turn right after the traffic signal turns green	Turn right as soon as the light turns green.	Smooth right turn on green light.
Normal 6	Crossing an intersection after the traffic signal turns green	Accelerate through the intersection as soon as the light turns green.	Accelerate smoothly through the intersection when the light turns green.
Normal 7	Straight-road cruising	Maintain a high traveling speed and keep a small safe distance from the vehicle in front of you.	Maintain a steady driving speed and keep a large safe distance from the vehicle in front of you
Normal 8	Crossing an intersection after the traffic signal turns green	Accelerate through the intersection as soon as the light turns green.	Accelerate smoothly through the intersection when the light turns green
Hazard 3	Sudden braking by the vehicle ahead	The system decelerates quickly to prevent collisions.	The system decelerates smoothly and maintains a safe following distance.

**FIGURE 4:**
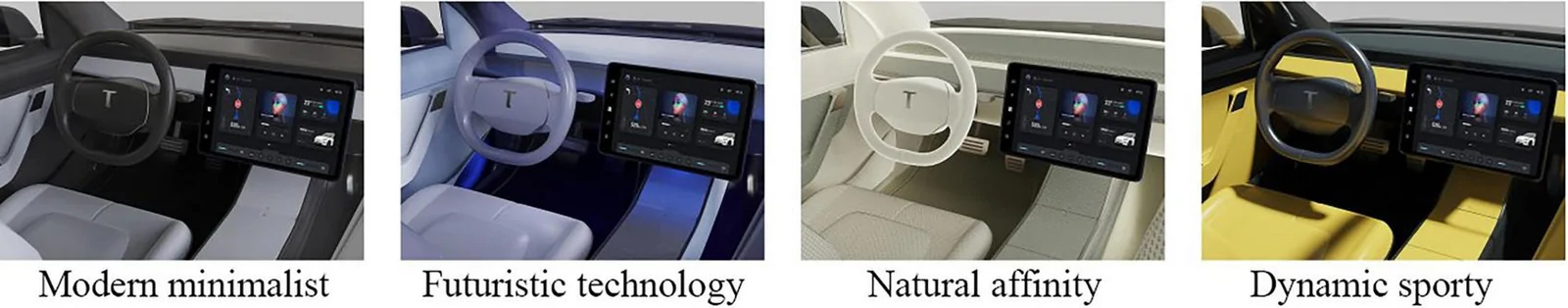
Autonomous vehicle interior design styles.

For the vehicle interior stimuli, four interior design styles identified through the pilot study were retained for the formal study: modern minimalist, futuristic technology-oriented, natural and inviting, and dynamic sporty. The corresponding three-dimensional vehicle interior models were developed by the authors and rendered using KeyShot. Materials, lighting conditions, surface treatments, and camera angles were adjusted to convey the intended visual characteristics of each style, as shown in Figure 4. After viewing each rendering, participants rated the extent to which it exemplified its intended interior design style category.

**FIGURE 5:**
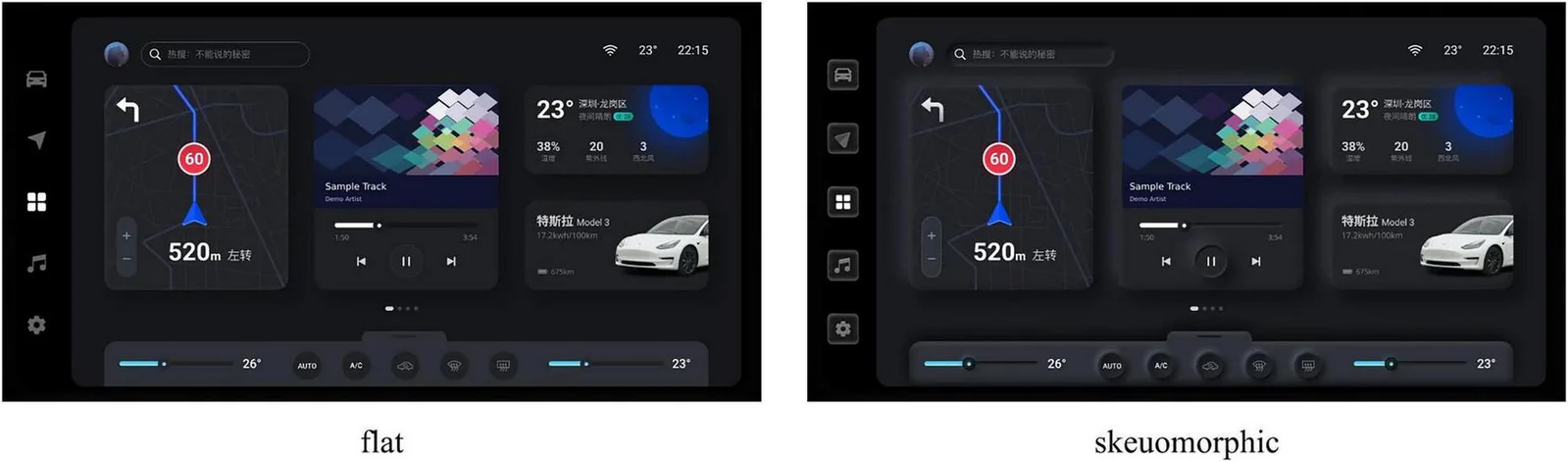
Flat and skeuomorphic HMI styles.

For the HMI stimuli, two styles identified through the pilot study—flat and skeuomorphic—were retained for the formal study. The corresponding interface images were created by the authors using Figma. To enhance comparability, the two interfaces presented the same information content, layout structure, and principal functional elements while differing in stylistic presentation, as shown in Figure 5. After viewing each interface, participants rated the extent to which it exemplified its intended HMI style category.

In the third stage, participants completed the measures of trust, PBC, and behavioral intention toward AVs. These measures were presented in questionnaire sections or pages separate from the stimulus-specific typicality evaluations and used different instructions and response anchors. The order of items within the relevant questionnaire sections was randomized to reduce order and context effects.

Several procedural measures were therefore incorporated to reduce potential common method bias. Participation was anonymous, participants were informed that there were no correct or incorrect answers, the stimulus evaluations and psychological measures were separated into different questionnaire sections or pages, and different task instructions and response anchors were used for the two types of measurement. Participants also completed the stimulus-specific evaluations before responding to the trust, PBC, and behavioral intention scales. The entire procedure took approximately 8–10 min to complete.

#### 3.2.4 Data analysis

Structural equation modeling (SEM) was conducted using Mplus version 8.3. The primary analyses used maximum likelihood (ML) estimation, with the nine five-point indicators of PBC, trust, and behavioral intention treated as continuous variables and the IVSTI entered as a continuous observed composite predictor. Univariate skewness and kurtosis were examined before model estimation. No item-level data were missing among the 202 retained cases.

First, confirmatory factor analysis (CFA) was conducted to evaluate the three-factor measurement model comprising perceived behavioral control (PBC), trust, and behavioral intention (Shojaei, 2024). The In-Vehicle Style Typicality Index (IVSTI) was excluded from the CFA because it was operationalized as an observed composite index rather than as a reflective latent construct. The measurement model was assessed using standardized factor loadings, composite reliability, average variance extracted (AVE), and discriminant-validity criteria. Convergent validity was evaluated using standardized factor loadings and AVE, whereas discriminant validity was assessed using the Fornell–Larcker criterion (Fornell and Larcker, 1981) and the heterotrait–monotrait ratio of correlations (HTMT; Henseler et al., 2015). Overall model fit was evaluated using the chi-square statistic, comparative fit index (CFI), Tucker–Lewis index (TLI), root mean square error of approximation (RMSEA), and standardized root mean square residual (SRMR).

Potential common method variance was assessed because the focal variables were obtained from the same respondents within a single survey session. In addition to the procedural controls described in Section 3.2.3, two CFA-based assessments were conducted. First, a one-factor model in which all nine PBC, trust, and behavioral-intention items loaded on a single factor was compared with the hypothesized three-factor measurement model. A substantially poorer fit of the one-factor model would indicate that the covariance among the reflective indicators could not be adequately represented by a single general factor. Second, an orthogonal common method factor with equal loadings across the nine reflective indicators was added to the model as a sensitivity analysis. The method factor was specified as orthogonal to PBC, trust, and behavioral intention, and the equal-loading constraint was used to provide a parsimonious representation of a uniform response tendency across the three scales. The IVSTI remained an observed composite index and was not modeled as either a reflective construct or an indicator of the method factor. To assess the robustness of the structural results, the six focal standardized path estimates were re-estimated in the common-method-factor model and compared with those from the primary structural model.

Second, the structural model was estimated with the IVSTI specified as an observed exogenous composite predictor and PBC, trust, and behavioral intention specified as latent endogenous constructs. The model included paths from the IVSTI to PBC, trust, and behavioral intention; from PBC to trust and behavioral intention; and from trust to behavioral intention. The paths from the IVSTI to the three endogenous constructs were used to evaluate H1a–H1c, while the remaining paths represented the component associations underlying the indirect pathways proposed in H2–H4. Collinearity among the three predictors of behavioral intention was also examined using variance inflation factors calculated from the model-implied predictor correlation matrix.

Third, the indirect associations between the IVSTI and behavioral intention were examined through PBC, through trust, and through the theoretically ordered sequence of PBC and trust. Bias-corrected 95% bootstrap confidence intervals were calculated using 5,000 bootstrap samples (Shojaei et al., 2026b). An indirect association was considered statistically supported when its confidence interval did not include zero. Given the cross-sectional design, the indirect effects were interpreted as statistical associations consistent with the hypothesized ordering rather than as evidence of temporal precedence or causality.

Finally, descriptive statistics and zero-order correlations were calculated for the eight typicality ratings, the three domain scores, and the IVSTI. To examine the domain-level pattern underlying the aggregate IVSTI results, automated driving-style typicality, interior design-style typicality, and HMI-style typicality were first tested in separate models and were subsequently entered simultaneously as correlated observed predictors. The measurement specification and the structural relations among PBC, trust, and behavioral intention were retained from the primary model. Alternative structural specifications included a parallel mediation model and an alternative serial model in which trust preceded PBC. Robustness was further examined using maximum likelihood estimation with robust standard errors (MLR) and by re-estimating the model using weighted least squares mean- and variance-adjusted estimation (WLSMV), with the nine five-point reflective indicators treated as ordered categorical variables and the IVSTI retained as a continuous observed predictor.

## 4 Results

Before model estimation, univariate skewness and kurtosis were examined. The absolute skewness and kurtosis values did not exceed 1.242 and 2.512, respectively, indicating no severe univariate nonnormality. The mean scores for the eight typicality ratings ranged from 3.861 to 4.347, with standard deviations ranging from 0.652 to 0.872. The mean domain scores were 4.131 (SD = 0.614) for automated driving-style typicality, 4.172 (SD = 0.584) for interior design-style typicality, and 3.923 (SD = 0.711) for HMI-style typicality. The mean IVSTI score was 4.075 (SD = 0.509). Descriptive statistics and zero-order correlations for the eight ratings, three domain scores, and the IVSTI are reported in Supplementary Table 2.

### 4.1 Measurement model

Confirmatory factor analysis (CFA) was conducted to evaluate the measurement properties of perceived behavioral control (PBC), trust, and behavioral intention. The In-Vehicle Style Typicality Index (IVSTI) was excluded from the CFA because it was operationalized as an observed composite index rather than as a reflective latent construct. The hypothesized three-factor measurement model demonstrated good fit to the data: χ^2^(24) = 34.038, *p* = 0.084, χ^2^/df = 1.418, CFI = 0.990, TLI = 0.985, RMSEA = 0.046, and SRMR = 0.029. The 90% confidence interval for the RMSEA ranged from 0.000 to 0.078, indicating that the proposed measurement model adequately represented the observed data.

The standardized factor loadings, composite reliability, and convergent-validity results are presented in Table 5. The standardized factor loadings ranged from 0.688 to 0.910 and were all statistically significant (*p* < 0.001), exceeding the recommended minimum value of 0.50. Composite reliability (CR) values ranged from 0.819 to 0.883, exceeding the recommended threshold of 0.70. The average variance extracted (AVE) values ranged from 0.602 to 0.716, exceeding the recommended threshold of 0.50. These results supported the reliability and convergent validity of the three reflective constructs.

**TABLE 5:** Composite reliability and convergent validity of the measurement model.

Construct	Item	Factor loading	S.E.	*z-*value	*P*-value	CR	AVE
PBC	PBC1	0.854	0.029	29.274	< 0.001	0.837	0.633
	PBC2	0.834	0.031	27.336	< 0.001		
	PBC3	0.688	0.043	16.085	< 0.001		
TR	TR1	0.858	0.025	34.627	< 0.001	0.883	0.716
	TR2	0.765	0.034	22.690	< 0.001		
	TR3	0.910	0.021	43.764	< 0.001		
BI	BI1	0.792	0.035	22.570	< 0.001	0.819	0.602
	BI2	0.820	0.033	24.634	< 0.001		
	BI3	0.712	0.042	16.845	< 0.001		

Discriminant validity was evaluated using both the Fornell–Larcker criterion and the heterotrait–monotrait ratio of correlations (HTMT). As shown in Table 6, the square root of the AVE for each construct, presented on the diagonal, was greater than its correlations with the other constructs. In addition, all HTMT values were below the recommended threshold of 0.85 (Henseler et al., 2015). These results supported the discriminant validity of PBC, trust, and behavioral intention.

**TABLE 6:** Discriminant validity assessment using the Fornell–Larcker criterion and HTMT ratios.

Construct	PBC	TR	BI
PBC	**0.796**	0.726	0.749
TR	0.734	**0.846**	0.706
BI	0.756	0.704	**0.776**

Bold diagonal values represent the square roots of the average variance extracted. Values below the diagonal represent latent construct correlations, whereas values above the diagonal represent HTMT ratios.

Potential common method variance was also assessed using CFA-based procedures. A one-factor model in which all nine PBC, trust, and behavioral-intention items loaded on a single factor demonstrated poor fit to the data: χ^2^(27) = 196.687, *p* < 0.001, CFI = 0.835, TLI = 0.780, RMSEA = 0.176, and SRMR = 0.070. The one-factor model fitted the data significantly less well than the hypothesized three-factor model, Δχ^2^(3) = 162.649, *p* < 0.001, indicating that the covariance among the reflective indicators could not be adequately represented by a single general factor. As an additional sensitivity analysis, an orthogonal common method factor with equal loadings across the nine reflective indicators was added to the model. After inclusion of the method factor, the maximum absolute change across the six focal standardized structural estimates was < 0.001, and the statistical significance and substantive interpretation of all focal paths remained unchanged. These findings did not indicate that a dominant uniform method factor materially distorted the principal associations, although residual shared method variance, including shared-method effects involving the IVSTI ratings, cannot be completely excluded.

Taken together, the results supported the reliability, convergent validity, and discriminant validity of the measurement model. The additional common method variance assessments further suggested that the observed associations were unlikely to be predominantly attributable to a single uniform method factor across the nine reflective indicators, although these assessments did not rule out shared-method effects involving the IVSTI ratings. The measurement model was therefore considered adequate for the subsequent structural and indirect-effect analyses.

### 4.2 Structural model

The hypothesized structural model was subsequently estimated with the In-Vehicle Style Typicality Index (IVSTI) specified as an observed exogenous composite predictor and PBC, trust, and behavioral intention specified as latent endogenous constructs. The model demonstrated an acceptable fit to the data: χ^2^(30) = 41.093, *p* = 0.085, χ^2^/df = 1.370, CFI = 0.990, TLI = 0.984, RMSEA = 0.043, and SRMR = 0.028.

The standardized path estimates are presented in Table 7. The IVSTI was positively associated with PBC (β = 0.341, *p* < 0.001), supporting H1a. The association between the IVSTI and trust was not statistically significant (β = 0.081, *p* = 0.183); therefore, H1b was not supported. The IVSTI was positively associated with behavioral intention (β = 0.185, *p* = 0.023), supporting H1c. The remaining structural paths were also statistically significant. PBC was positively associated with trust (β = 0.706, *p* < 0.001) and behavioral intention (β = 0.480, *p* < 0.001), while trust was positively associated with behavioral intention (β = 0.292, *p* = 0.009).

**TABLE 7:** Standardized structural path estimates and hypothesis-testing results.

Structural path	Std. Est. (β)	S.E.	*z-*value	*P*-value	Hypothesis result
IVSTI → PBC (H1a)	0.341	0.07	4.891	< 0.001	Supported
IVSTI → TR (H1b)	0.081	0.061	1.331	0.183	Not supported
IVSTI → BI (H1c)	0.185	0.082	2.268	0.023	Supported
PBC → TR	0.706	0.065	10.848	< 0.001	–
PBC → BI	0.480	0.118	4.049	< 0.001	–
TR → BI	0.292	0.111	2.625	0.009	–

Collinearity diagnostics were also examined for the three predictors of behavioral intention. The variance inflation factors were 1.15 for the IVSTI, 2.22 for PBC, and 2.19 for trust. These values were below conventional thresholds for problematic multicollinearity, indicating that multicollinearity did not materially compromise the simultaneous estimation of the predictors’ associations with behavioral intention.

The model explained 11.7% of the variance in PBC, 54.5% of the variance in trust, and 65.1% of the variance in behavioral intention. Domain-level analyses examining driving-style, interior-design-style, and HMI-style typicality separately and simultaneously are reported in section 4.4.1.

### 4.3 Indirect effects

The results of the indirect-effect analysis are presented in Table 8. Based on 5,000 bias-corrected bootstrap samples, the IVSTI had a statistically significant total association with behavioral intention [*B* = 0.456, *p* < 0.001, 95% CI (0.270, 0.636)]. After accounting for PBC and trust, the direct association between the IVSTI and behavioral intention remained statistically significant [*B* = 0.191, *p* = 0.019, 95% CI (0.037, 0.352)].

**TABLE 8:** Bias-corrected bootstrap estimates of direct and indirect effects.

Effect	Path	Unstd. Est. (B)	Boot S.E.	*P*-value	Bias-corrected 95% bootstrap CI
Total effect	IVSTI → BI	0.456	0.095	< 0.001	[0.270, 0.636]
Indirect effect	IVSTI → PBC → BI (H2)	0.169	0.059	0.004	[0.080, 0.323]
	IVSTI → TR → BI (H3)	0.024	0.022	0.263	[−0.006, 0.082]
	IVSTI → PBC → TR → BI (H4)	0.073	0.033	0.029	[0.025, 0.161]
Direct effect	IVSTI → BI	0.191	0.081	0.019	[0.037, 0.352]

Confidence intervals were calculated using 5,000 bootstrap samples.

Regarding the specific indirect associations, the indirect association between the IVSTI and behavioral intention through trust was not statistically significant [*B* = 0.024, 95% CI (−0.006, 0.082)], as the confidence interval included zero. Therefore, H3 was not supported. In contrast, the indirect association through PBC was statistically significant [*B* = 0.169, 95% CI (0.080, 0.323)], supporting H2. The serial indirect association through PBC and trust was also statistically significant [*B* = 0.073, 95% CI (0.025, 0.161)], supporting H4.

**FIGURE 6:**
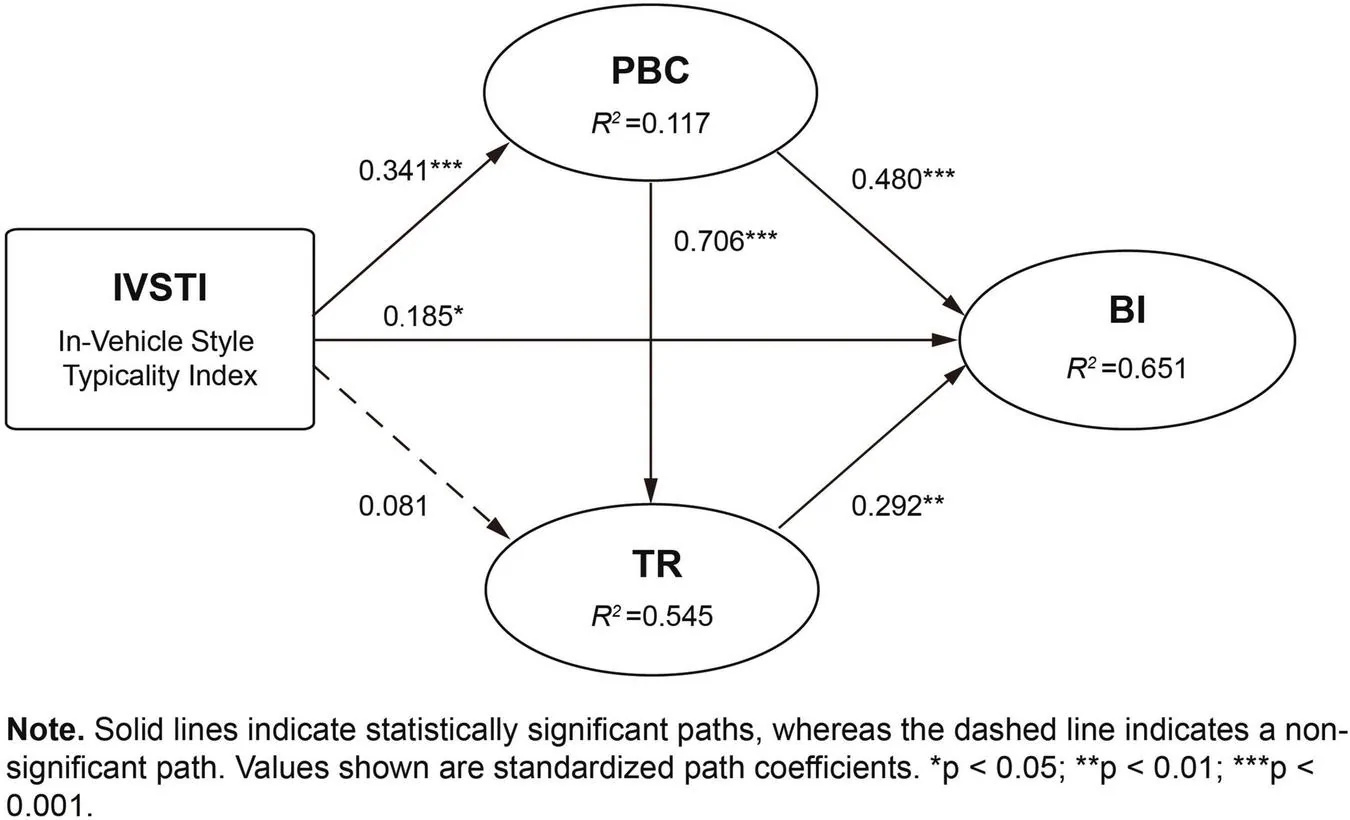
Structural model with standardized path coefficients.

Taken together, the IVSTI was associated with behavioral intention both directly and indirectly, with PBC accounting for the largest specific indirect effect. The serial indirect association through PBC and trust was also statistically significant. The estimated model is illustrated in Figure 6.

### 4.4 Additional analyses

#### 4.4.1 Domain-level analyses

To examine whether the associations observed for the aggregate IVSTI were concentrated in a particular stylistic domain, automated driving-style typicality, interior design-style typicality, and HMI-style typicality were first examined in separate models and were subsequently entered simultaneously as correlated observed predictors. In each model, the measurement specification and the structural relations among PBC, trust, and behavioral intention were retained from the main model. Table 9 presents the standardized path estimates from the separate and simultaneous domain-level models.

**TABLE 9:** Domain-level associations in the separate and simultaneous models.

Outcome	Predictor	Separate model β (S.E.)	*P*-value	Simultaneous model β (S.E.)	*P*-value
PBC	Automated driving-style typicality	0.247 (0.087)	0.004	0.133 (0.098)	0.175
	Interior design-style typicality	0.269 (0.075)	< 0.001	0.081 (0.105)	0.444
	HMI-style typicality	0.300 (0.072)	<0.001	0.217 (0.094)	0.022
Trust	Automated driving-style typicality	0.058 (0.055)	0.292	0.062 (0.070)	0.370
	Interior design-style typicality	0.026 (0.060)	0.667	−0.058 (0.081)	0.473
	HMI-style typicality	0.095 (0.064)	0.138	0.106 (0.073)	0.148
Behavioral intention	Automated driving-style typicality	0.159 (0.069)	0.020	0.101 (0.077)	0.191
	Interior design-style typicality	0.163 (0.077)	0.035	0.092 (0.088)	0.295
	HMI-style typicality	0.106 (0.081)	0.193	0.037 (0.081)	0.651

All three separate models demonstrated acceptable fit to the data (CFI = 0.986–0.993, TLI = 0.979–0.989, RMSEA = 0.036–0.049, and SRMR = 0.028–0.030). When examined separately, automated driving-style typicality (β = 0.247, *p* = 0.004), interior design-style typicality (β = 0.269, *p* < 0.001), and HMI-style typicality (β = 0.300, *p* < 0.001) were each positively associated with PBC. Automated driving-style typicality (β = 0.159, *p* = 0.020) and interior design-style typicality (β = 0.163, *p* = 0.035) were also positively associated with behavioral intention, whereas the corresponding association for HMI-style typicality was not statistically significant (β = 0.106, *p* = 0.193). None of the three domain scores was significantly associated with trust when examined separately.

The simultaneous domain-level model demonstrated good fit to the data: χ^2^(42) = 52.559, *p* = 0.127, χ^2^/df = 1.251, CFI = 0.990, TLI = 0.985, RMSEA = 0.035, and SRMR = 0.027. After accounting for the overlap among the three domains, HMI-style typicality remained positively associated with PBC (β = 0.217, *p* = 0.022), whereas the unique associations of automated driving-style typicality (β = 0.133, *p* = 0.175) and interior design-style typicality (β = 0.081, *p* = 0.444) with PBC were not statistically significant. None of the three domain scores showed a statistically significant unique association with trust or behavioral intention.

The structural relations among the endogenous constructs remained consistent with those observed in the main model. PBC was positively associated with trust (β = 0.703, *p* < 0.001) and behavioral intention (β = 0.478, *p* < 0.001), and trust was positively associated with behavioral intention (β = 0.298, *p* = 0.009). The simultaneous model explained 12.0% of the variance in PBC, 55.0% of the variance in trust, and 65.3% of the variance in behavioral intention.

Taken together, the domain-level analyses revealed both shared and domain-specific patterns. All three domain scores were positively associated with PBC when examined separately, whereas HMI-style typicality showed the only statistically significant unique association with PBC after the overlap among the domains was taken into account.

#### 4.4.2 Alternative-model and estimator-sensitivity analyses

Two alternative structural specifications were estimated using the same ML estimator and 5,000-sample bias-corrected bootstrap procedure. The parallel mediation model and the alternative serial model in which trust preceded PBC yielded the same global fit indices as the primary model: χ^2^(30) = 41.093, *p* = 0.085, CFI = 0.990, TLI = 0.984, RMSEA = 0.043, and SRMR = 0.028.

In the parallel mediation model, the IVSTI was positively associated with both PBC (β = 0.341, *p* < 0.001) and trust (β = 0.322, *p* < 0.001). The indirect associations with behavioral intention through PBC [*B* = 0.169, 95% CI (0.080, 0.323)] and trust [*B* = 0.097, 95% CI (0.028, 0.211)] were both statistically significant. In the alternative serial model, the IVSTI was positively associated with trust (β = 0.322, *p* < 0.001), and trust was positively associated with PBC (β = 0.696, *p* < 0.001). The serial indirect association through trust followed by PBC was also statistically significant [*B* = 0.111, 95% CI (0.054, 0.211)]. These results indicate that the cross-sectional data did not empirically distinguish the temporal ordering of PBC and trust.

Estimator-sensitivity analyses were also conducted. The model estimated using MLR demonstrated an acceptable fit to the data: χ^2^(30) = 41.366, *p* = 0.081, CFI = 0.989, TLI = 0.983, RMSEA = 0.043, and SRMR = 0.028. When the nine five-point reflective indicators were treated as ordered categorical variables and the model was estimated using WLSMV, the fit indices were χ^2^(30) = 58.080, *p* = 0.002, CFI = 0.991, TLI = 0.986, RMSEA = 0.068, and SRMR = 0.041. Under both estimators, the IVSTI remained significantly associated with PBC and behavioral intention but not with trust, while the associations among PBC, trust, and behavioral intention remained statistically significant. The indirect association through PBC and the serial indirect association through PBC and trust also remained statistically significant, whereas the indirect association through trust alone remained nonsignificant. Thus, the principal findings were not materially altered by the choice of estimator or the treatment of the five-point reflective indicators.

## 5 Discussion

### 5.1 Theoretical and practical implications

Previous studies have shown that individual design-related factors, including automated driving style and HMI design, are associated with users’ evaluations and acceptance of autonomous vehicles (Large et al., 2019; Lu et al., 2022; Ma and Zhang, 2021; Ruijten et al., 2018). From an occupant-centered perspective, however, automated driving behavior, interior design, and HMI presentation are encountered within the same autonomous journey: occupants experience how the vehicle moves and responds, how the cabin environment is presented, and how the system communicates through the HMI. These dynamic, environmental, and interactional sources of stylistic information provide complementary inputs to the broader in-vehicle experience. Considering them within a common framework therefore extends autonomous vehicle acceptance research beyond isolated design domains. This common framework does not imply that the three domains are interchangeable manifestations of a single underlying construct. Rather, by examining perceived typicality within each domain and summarizing the domain-level scores through the observed composite IVSTI, the present study provides an aggregate perspective while preserving their conceptual distinctiveness.

The associations of the IVSTI with PBC and behavioral intention, together with the significant indirect association through PBC, highlight perceived behavioral control as an important statistical pathway in the proposed model. Importantly, the IVSTI captures how closely the presented cues correspond to their intended stylistic categories rather than users’ preferences for particular styles. One possible interpretation is that stronger perceived correspondence may be associated with greater interpretability of the vehicle’s behavior, cabin environment, and system communication, as well as with a stronger sense that the vehicle is understandable and manageable. This interpretation is consistent with previous evidence linking perceived capability and manageability with behavioral intentions toward technology use (Ajzen, 1991; Luo et al., 2023; Molinillo et al., 2024). The statistically significant direct association with behavioral intention after PBC and trust were included in the model further suggests that these two internal evaluations do not fully account for the observed association between the IVSTI and behavioral intention. Additional evaluative pathways were not examined in the present model.

The domain-level analyses further qualified the aggregate IVSTI findings. When examined separately, automated driving-style, interior design-style, and HMI-style typicality were each positively associated with PBC, while automated driving-style and interior design-style typicality were also positively associated with behavioral intention. When the three domain scores were entered simultaneously, however, only HMI-style typicality retained a statistically significant unique association with PBC, and none showed a statistically significant unique association with trust or behavioral intention. Thus, the aggregate association with PBC was not confined to a single domain in the separate models, although HMI-style typicality showed the clearest unique association after the overlap among the three domains was taken into account. These results indicate that the three domains contribute differentiated rather than uniform patterns within the broader in-vehicle experience.

The absence of a statistically significant direct association between the IVSTI and trust, together with the nonsignificant indirect association through trust alone, suggests that perceived typicality and perceived trustworthiness represent different evaluations. The typicality ratings assessed how closely each stimulus corresponded to its intended stylistic category rather than whether that category was perceived as safe, dependable, or desirable. For example, aggressive and defensive driving behaviors may both be perceived as highly typical of their respective categories while conveying different implications for reliance. Similarly, the interior and HMI styles represent categorical alternatives rather than higher and lower levels of trustworthiness. Consequently, a high IVSTI score indicates strong average correspondence between the presented cues and their intended categories, but it does not necessarily indicate that those cues consistently communicate safety or reliability.

Trust in autonomous vehicles may instead depend on a broader range of evaluations, including technical competence, safety performance, institutional safeguards, prior experience, and social information, rather than on stylistic cues alone (Riegelsberger et al., 2005). More tentatively, stylistic information encountered within the in-vehicle experience may relate differently to cognitive and affective bases of trust. The current trust items emphasize system safety, safeguards, and overall trustworthiness, but they do not permit a clear separation between cognitive and affective bases of trust. Consequently, the present data cannot determine whether stylistic information is more closely related to capability-based understanding than to emotional reassurance. The findings therefore do not imply that stylistic information is irrelevant to trust, but they indicate that perceived typicality alone is insufficient to account for overall trust in autonomous vehicles.

The serial indirect association in the primary model was consistent with the hypothesized ordering of PBC followed by trust. However, the parallel mediation model and the alternative serial model in which trust preceded PBC also produced statistically significant indirect associations and showed fit equivalent to that of the primary model. The cross-sectional data therefore did not distinguish whether PBC precedes trust, trust precedes PBC, or the two operate as parallel internal evaluations. Accordingly, the primary serial association should be interpreted as one theoretically ordered statistical representation that is consistent with the data rather than as evidence of a confirmed psychological sequence. The principal pattern of findings also remained unchanged when the model was re-estimated using MLR and WLSMV, indicating that the main conclusions were not materially dependent on the estimator or on treating the five-point reflective indicators as continuous rather than ordered categorical variables.

The study also provides two practical implications. First, automated driving behavior, interior design, and HMI presentation should be considered as distinct components encountered within the same occupant experience rather than as interchangeable design domains. Designers may consider automated driving behavior, interior design, and HMI presentation together when planning the broader occupant experience, while recognizing that the three domains convey distinct forms of stylistic information. The unique association between HMI-style typicality and PBC in the simultaneous domain-level model further suggests that HMI presentation may warrant particular attention when supporting occupants’ perceptions that autonomous vehicle use is understandable and manageable. However, the present findings do not establish that stylistic consistency or coordination across the three domains increases acceptance; such cross-domain relationships would need to be examined directly using integrated vehicle configurations.

Second, the lack of a statistically significant direct association between the IVSTI and trust indicates that stylistic presentation should not be treated as a substitute for information about system performance and safety. Trust-related design and communication strategies may therefore need to combine appropriate stylistic presentation with clear information about system capabilities, reliability, safety performance, operational limitations, and available safeguards.

### 5.2 Limitations and future research

This study has several limitations. First, although automated driving style, interior design style, and HMI style were considered within an occupant-centered view of the broader in-vehicle experience, the study did not directly examine coordination or interaction among the three domains or compare specific style-category combinations. The IVSTI provides an aggregate summary of perceived typicality across the domains, but it does not represent the stylistic coherence of an integrated vehicle configuration. Future research could therefore examine whether particular combinations of automated driving behavior, interior design, and HMI presentation show complementary or conflicting associations with PBC, trust, and behavioral intention.

Second, the study relied on simulated automated driving videos and static vehicle interior and HMI images. Although these materials allowed the intended stylistic characteristics to be presented in a controlled manner, they may not fully capture the interactivity, physical sensation, and uncertainty of real-world autonomous driving. Moreover, the pilot sorting exercise used candidate images to identify the target interior and HMI style categories, whereas the final researcher-created stimuli were not independently pretested before the main study. The typicality ratings obtained from the main sample therefore assessed perceived correspondence with the intended categories but did not constitute independent validation of the final stimuli. Future studies could independently pretest newly created stimuli and employ immersive virtual reality, high-fidelity simulators, prototype vehicles, or field evaluations.

Third, the cross-sectional and self-reported data limit temporal and causal interpretation. In particular, the ordered pathway involving PBC and trust represents a theoretically specified statistical association rather than a confirmed process in which perceived control necessarily precedes trust. The statistically supported parallel and trust-first alternative models further indicate that the present data cannot determine the temporal ordering of the two constructs. Although procedural controls and CFA-based assessments did not indicate that a dominant uniform method factor materially distorted the findings, shared-method effects involving the IVSTI ratings cannot be completely excluded. In addition, the trust items emphasized system safety, safeguards, and overall trustworthiness but did not permit a clear separation between cognitive and affective bases of trust. Longitudinal studies, controlled experiments, behavioral indicators, and more differentiated trust measures are needed to clarify how PBC and trust are related over time.

Finally, the sample consisted predominantly of young adults with relatively limited driving experience, which may restrict the generalizability of the findings. Although younger adults represent an important group of prospective SAE Level 5 AV users, age, driving experience, and cultural background may shape expectations of vehicle behavior and evaluations of control and trust. Future research should therefore examine whether the observed associations generalize to older adults, more experienced drivers, and users from different cultural contexts.

## 6 Conclusion

This study examined the direct association between the In-Vehicle Style Typicality Index (IVSTI) and behavioral intention toward autonomous vehicles, as well as the indirect associations through perceived behavioral control (PBC) and trust. The IVSTI summarized perceived typicality across three conceptually distinct domains that occupants encounter within the in-vehicle experience: automated driving style, interior design style, and HMI style.

The results showed that the IVSTI was positively associated with both PBC and behavioral intention, and the indirect association through PBC was statistically significant. By contrast, the IVSTI did not show a statistically significant direct association with trust, and the indirect association through trust alone was not statistically significant. The serial indirect association through PBC and trust was statistically significant; however, the parallel mediation model and the alternative serial model in which trust preceded PBC were also statistically supported. The cross-sectional data therefore did not establish a unique ordering between PBC and trust. The principal pattern of findings remained stable in the MLR and WLSMV sensitivity analyses.

These findings extend autonomous vehicle acceptance research by examining the perceived typicality of stylistic cues across automated driving behavior, interior design, and HMI presentation within an occupant-centered framework that encompasses the three domains while preserving their conceptual distinctiveness. The domain-level analyses further showed that all three domain scores were positively associated with PBC when examined separately, whereas only HMI-style typicality retained a statistically significant unique association with PBC when the three domains were examined simultaneously. They also identify PBC as an important statistical pathway linking the perceived typicality of in-vehicle stylistic cues with behavioral intention, particularly through the specific indirect association through PBC. At the same time, the findings do not indicate that the perceived typicality of in-vehicle stylistic cues alone accounts for trust in autonomous vehicles. Trust-related design and communication strategies may therefore need to incorporate information about system capabilities, safety performance, reliability, and operational limitations in addition to stylistic presentation.

## Data Availability

The raw data supporting the conclusions of this article will be made available by the authors, without undue reservation.
